# Discovery of the Nicotinic Receptor Toxin Anabaseine in a *Polystiliferan* Nemertean

**DOI:** 10.3390/toxins15010046

**Published:** 2023-01-05

**Authors:** William R. Kem, James R. Rocca, Jodie V. Johnson, Juan Junoy

**Affiliations:** 1 Department of Pharmacology and Therapeutics, University of Florida College of Medicine, 1200 Newell Drive, Gainesville, FL 32610, USA; 2 AMRIS, McKnight Brain Institute, University of Florida, Gainesville, FL 32610, USA; 3 Department of Chemistry, University of Florida, Gainesville, FL 32611, USA; 4 Departamento de Ciencias de la Vida, Universidad de Alcalá, 28805 Alcalá de Henares, Spain

**Keywords:** anabaseine, anabasine, ehrlich reagent, nemertean, nemertine, nicotinic acetylcholine receptor, toxin, venom

## Abstract

Nemerteans (also called Nemertines) are a phylum of predominantly marine worms that use toxins to capture prey and to defend themselves against predators. Hoplonemerteans have a proboscis armed with one or more stylets used in prey capture and are taxonomically divided into Order Monostilifera, whose members possess a single large proboscis stylet, and Order Polystilifera, whose members have multiple small stylets. Many monostiliferans contain alkaloidal toxins, including anabaseine, that stimulate and then desensitize nicotinic acetylcholine receptors that are present in all animals. These compounds also interact with pyridyl chemoreceptors in crustaceans, reducing predation and larval settlement. Anabaseine has been a lead compound in the design of alpha7 nicotinic acetylcholine receptor agonists like GTS-21 (also called DMXBA) to treat disorders of cognition such as Alzheimer’s disease and schizophrenia. These drug candidates also display anti-inflammatory activities of potential medical importance. Most polystiliferans live deep in open oceans and are relatively inaccessible. We fortunately obtained two live specimens of a large benthic polystiliferan, *Paradrepanophorus crassus* (*Pc*), from the coast of Spain. MS and NMR analyses of the Ehrlich’s reagent derivative allowed identification of anabaseine. A spectrophotometric assay for anabaseine, also based on its reaction with Ehrlich’s reagent, revealed high concentrations of anabaseine in the body and proboscis. Apparently, the biosynthetic mechanism for producing anabaseine was acquired early in the evolution of the Hoplonemertea, before the monostiliferan-polystiliferan divergence.

## 1. Introduction

**Figure 1:**
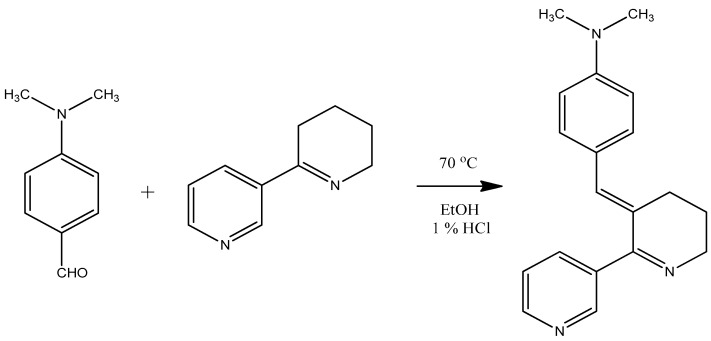
Reaction of Ehrlich’s reagent (p-dimethylaminobenzaldehyde) with anabaseine. The product, DMAB-anabaseine, at 3.0 µg/mL has a high visible light absorbance of 1.00 at 490 nm in ethanol containing 1% concentrated HCl, permitting detection of very small amounts of anabaseine in tissue extracts and on thin layer chromatograms (adapted from [7]).

Based on the structure of the proboscis, it was surmised that hoplonemertean (also called hoplonemertine) worms use venom to capture their prey [1,2]. The presence of toxic substances in nemerteans was discovered in the 1930s. The Belgian pharmacologist Bacq found that crude extracts of *Amphiporus lactifloreus*, a relatively common hoplonemertean along north Atlantic coasts, stimulated neuromuscular and autonomic ganglionic synapses like acetylcholine, but were not inactivated by high alkaline pH [3,4]. An attempt to isolate the active compound, named “amphiporine,” by solvent extraction and picric acid crystallization methods widely used in isolating plant alkaloids was only partially successful due to the availability of only a small number of worms and to difficulties in obtaining a crystalline salt suitable for structure analysis. Nevertheless, King [5] demonstrated that “amphiporine” behaved like a weakly basic alkaloid. Thirty years later a compound with nicotinic actions on vertebrate skeletal muscle was isolated from the wandering nemertean, *Paranemertes peregrina (Pp)*, which is widely distributed along north Pacific coastal waters of Asia and America. Anabaseine (Figure 1) was isolated as a free base using aluminum oxide thin layer, preparative layer and ultimately column chromatography [6]. A chemical assay based on Ehrlich’s reagent (p-dimethylaminobenzaldehyde, Figure 1) facilitated the initial purification and identification of anabaseine [6] and also its detection in other nemerteans [7,8]. Anabasine, a tree tobacco alkaloid, resembles anabaseine but lacks its imine bond. Spath and Mamoli, tobacco alkaloid chemists at the University of Vienna, first prepared anabaseine as an intermediate in their synthesis of anabasine [9]. Using the Ehrlich and other thin-layer chromatographic spot reagents, it was found that a number of hoplonemertean species possess anabaseine, anabasine [7,8] and/or related pyridyl compounds like 2,3′-bipyridyl [10], nemertelline [10], 3-methyl-2,3′-bipyridyl [11], and isoanatabine [12,13], suggesting that this family of natural products appeared early in monostyliferan evolution. Fortunately, the Ehrlich reagent based spectrophotometric assay for anabaseine has allowed its presence to be detected in even small nemerteans, as most species are small and difficult to collect in sufficient numbers for chemical (especially NMR) analyses. 

**Figure 2:**
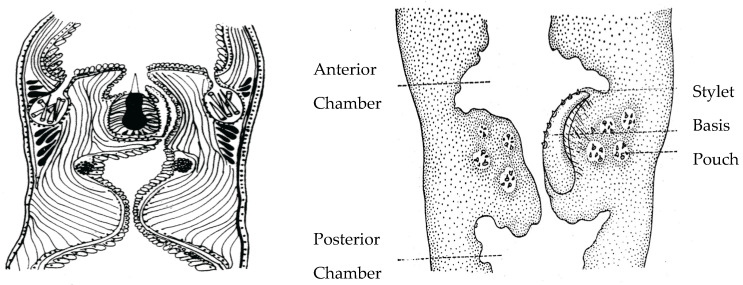
Hoplonemertean stylets. **Left**: Stylet apparatus of a monostiliferan hoplonemertean. **Right**: Stylet apparatus of a pelagic polystiliferan hoplonemertean. Top of each median proboscis shows anterior proboscis chamber, bottom shows posterior proboscis chamber. The polystiliferan stylet basis containing multiple small stylets is probably used to abrade or tear the prey’s integument, facilitating venom entry. Accessory stylets are synthesized and stored in nearby mineralizing gland pouches in both probosces.

Current understanding of nemertean phylogeny is incomplete compared with many animal phyla, in spite of systematic investigations by a number of expert systematists. Within the hoplonemertea Order Polystilifera has been less studied because most species live deep in the open ocean and are difficult to collect. Because they are considered a monophyletic group [14,15,16,17], i.e., they evolved from a common ancestor, it is of great interest to identify their toxins to understand how the hoplonemertean proboscis and its venom-producing and -injecting system evolved over time. Currently one can only speculate that the first stylets were small and relatively simple and that this primitive weapon was largely retained by polystiliferans which, instead of developing large single stylets as found in monostiliferans, developed a basis containing many small stylets (Figure 2).

**Figure 3:**
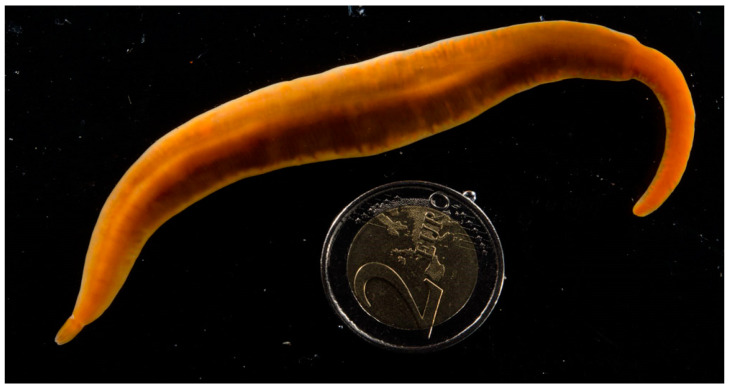
Color photograph of the living *Paradrepanophorus crassus* specimen used in this study. The head is at the bottom left side of the photograph. While spontaneously gliding in a dish of sea water it displayed a maximum length of approximately 25 cm and a maximum width of 1.5 cm. The specimen had a regenerated tail (top right) as revealed by the sudden change in its body width. A two Euro coin (26 mm diameter) was included to provide a scale.

This paper is the first report of the existence of anabaseine in a species belonging to Order Polystilifera. Over 100 polystiliferan hoplonemertean species have been described by taxonomists [14,15,16,17]. Most are fragile, bathypelagic animals living at great depths in the oceans of the world and are known from just a few specimens, often mutilated during their collection with conventional nets or dredges. However, there also are a small number (~30 species) of benthic polystiliferans that live in shallow coastal waters at the lowest part of the intertidal zone or in adjacent shallow waters. *Paradrepanophorus crassus* (abbreviated *Pc*), the subject of this paper, is one of these benthic species. *Pc* has been reported to occur on British, Irish and Spanish Atlantic coasts as well as in the Mediterranean Sea [18,19,20]. While the presence of numerous tiny stylets in the proboscis of polystiliferans has been noted, the only previous report of a toxin in this group was Bacq’s observation that an aqueous extract of this species displayed “amphiporine” activities, stimulating skeletal muscle and autonomic synapses [3,4]. Here, we report the presence of high concentrations of anabaseine in the body and proboscis of this species (Figure 3).

## 2. Results

### 2.1. Anabaseine Identification

**Figure 4:**
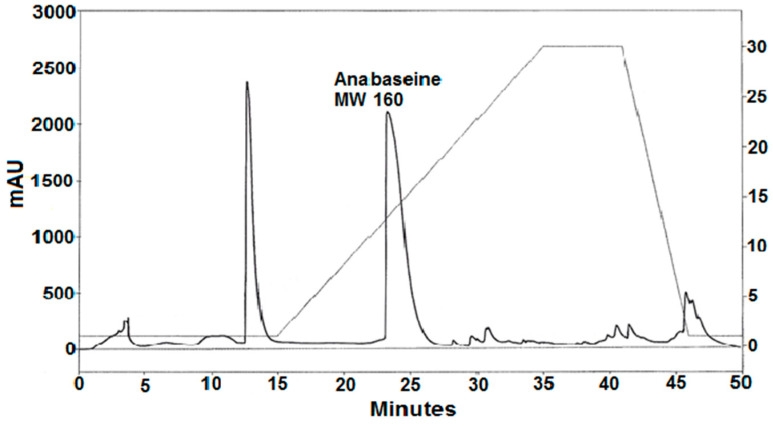
Silica gel HPLC separation of a crude methylene chloride extract of alkaloids from the *Pc* worm. A linear gradient of an increasing concentration (2 to 30%, shown in right y-axis) of isopropanol in hexane-0.2% triethylamine was used with a Beckmann silica gel analytical column at a flow rate of 1.5 mL/min. Absorbance at 260 nm was measured with a diode array detector.

A silica gel gradient HPLC separation of a portion of the basic methylene chloride phase is shown in Figure 4. Two major peaks eluting at 13 and 23 min were observed. The absorbance spectrum of the second peak contained two major peaks, at 230 and 260 nm with the absorbance of the 230 nm peak being ~1.5× larger than the 260 nm peak, similar to the spectrum for unionized anabaseine injected into the same column. The presumed anabaseine peak was collected, evaporated to dryness in a rotary evaporator and subjected to reverse phase C18 LC-Electrospray Ionization MS. Like synthetic anabaseine, the isolated compound produced LC-ESI-MS *m/z* 161 and *m/z* 179 ions which eluted simultaneously with the LC-UV absorbance peak, whose retention time was typical for anabaseine; the 161 peak corresponded to the cyclic iminium form of anabaseine and the 179 peak to the open-chain ammonium-ketone form of anabaseine [21,22].

If they were present, two other known hoplonemertean alkaloids (2,3′-bipyridyl [10] and 3-methyl-2,3′-bipyridyl [11]), being less basic and polar, would have eluted earlier than anabaseine, and three other more polar nemertean alkaloids (isoanatabine [12], anabasine [8] and nemertelline [10]) would have eluted after anabaseine. However, re-HPLC of the 13 min and post-15 min peak components shown in Figure 4 failed to reveal these compounds. Smaller amounts of other pyridyl compounds might be found in future studies when more worms become available.

**Figure 5:**
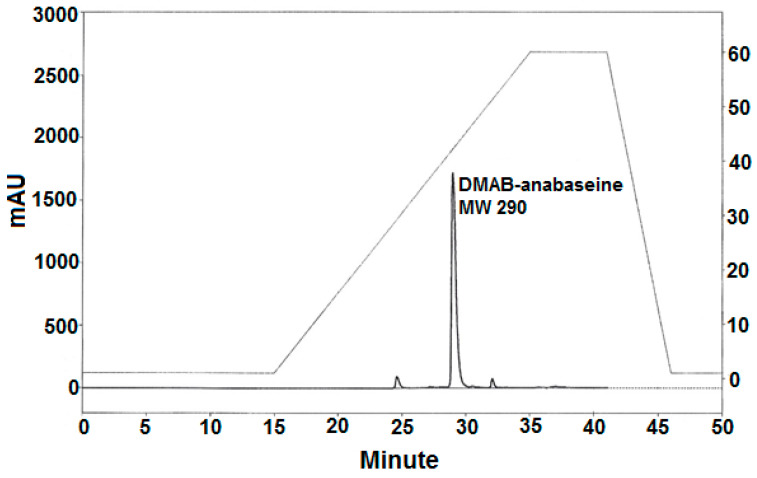
Normal phase HPLC separation of the Ehrlich reaction products from the crude ethanolic *Paradrepanophorus crassus* body extract. The same silica gel column was used as in the normal phase HPLC separation shown in Figure 4, but a steeper gradient (%B, shown by dashed line and right y-axis dimensions) of increasing proportion of isopropanol-0.1% triethylamine in hexane-0.1% triethylamine was necessary to elute the putative 3-(4-dimethylaminobenzylidene)-anabaseine which occurred at 29 min.

Most of the crude alkaline methylene chloride phase alkaloid sample was reacted with Ehrlich’s reagent under acidic conditions and subsequently purified on the same silica gel analytical HPLC column used to obtain the putative anabaseine component. The DMAB-derivative (Figure 5) eluted at a higher isopropanol concentration due to its higher polarity; synthetic DMAB-anabaseine eluted with an almost identical retention time (data not shown). A high resolution ESI-MS analysis of the DMAB-natural product resulted in an *m/z* 292.1811 [M+H]^+^ ion which was in good agreement (0.96 ppm) with the theoretical *m*/*z* 292.1808 [M+H]^+^ ion of the neutral elemental composition (C_19_H_21_N_3_), consistent with the structure of DMAB-anabaseine shown in Figure 1. 

The proton data (Table 1) extracted from a 300 MHz proton NMR spectrum of the HPLC-purified DMAB-derivative free base were essentially identical with the 600 MHz spectral data from the synthesized DMAB-anabaseine free base, completing the structure identification of the natural product. 

### 2.2. Anabaseine Concentrations in Pc Tissues

Anabaseine concentrations within the largest *Pc* worm are summarized in Table 2. Previously published results [7] for *Pp* obtained using the same procedure are also shown to facilitate comparisons between the two species. In both species most of the anabaseine was found in the body, suggesting that it is used as a chemical defense against potential predators. Unlike the *Pp* proboscis, which contained 27% of the total worm anabaseine, the anterior proboscis of *Pc* contained only 2% of the total anabaseine, and its anabaseine tissue concentration was approximately 50× less than in the *Pp* anterior proboscis. It is interesting that the *Pc* anterior and posterior proboscis concentrations are similar. In contrast, the *Pp* anterior proboscis has much higher anabaseine concentrations than the body proper or the posterior proboscis [7]. In early scientific speculations, before any toxins were discovered, it was assumed that a hoplonemertean’s venom would be stored in the lumen of the posterior proboscis so that it could be ejected more readily at sites of stylet puncture of the prey organism [1,2]. The differences between the *Pc* and *Pp* anabaseine distributions, if real, might relate to different methods of prey capture. *Pp* wraps its anterior proboscis around its annelid prey whereas *Pc* may be a suctorial predator of crustaceans, like the monostiliferous chevron hoplonemertean *Amphiporus angulatus* [10,11,12,13]. Suctorial species probably rely on rupturing the crustacean exoskeleton to allow toxin penetration. Clearly further analyses of proboscis alkaloid concentrations in different parts of hoplonemertean probosces are needed to understand the mechanics of toxin synthesis and envenomation and how it may vary with the species.

Our *Pc* anabaseine estimates, based on a single specimen, are less reliable than the *Pp* estimates, which are mean values based on three separate experiments, each with ten adult worms. Additionally, the *Pc* anabaseine estimates may be on the low side since there was a significant period of time (one month) between the initial preservation of the *Pc* parts and the anabaseine determinations. Additionally, the tissues were preserved and then mailed in pure ethanol rather than a more stabilizing mixture like ethanol containing 1% glacial acetic acid where pH < 3. Anabaseine is not very stable in the intermediate (3–9) pH range [21,22,23] where both unionized and ionized iminium as well as open chain forms co-exist, due to its tendency to polymerize and possible form Schiff base adducts with other molecules [23]. As more specimens of *Pc* become available it will be possible to extend the current observations and search for minor pyridine alkaloid constituents that were not detected here. 

## 3. Discussion

The body concentration of anabaseine in the large *Pc* specimen was one of the highest concentrations observed to date, but less than half the concentration in *Pp* [7,8]. A second *Pc* specimen of 0.4 g fresh weight was also collected. The total anabaseine concentration of this undissected worm was found to be 720 ug/g using the DMAB assay. It is possible that the lower concentrations of anabaseine in *Pc* relative to *Pp* was partly due to the lower body surface area: weight ratio of these large worms. It was previously shown that nemertean body toxins are localized in their integument [7,8,24]. The maximum length of the large *Pc* specimen was approximately 25 cm and its total fresh weight was 2.90 g. Assuming that the concentration of anabaseine is the same in the integumentary tissues of *Pc* as in *Pp*, and assuming a cylindrical form for both species, one calculates a surface area: fresh weight ratio of only 12 cm^2^/g for *Pc*, compared with a 36 cm^2^/g ratio for the *Pp* worms which averaged 15 cm length, 3 mm diameter and 0.4 g fresh weight. Thus, one would expect a 2–3 fold lower body anabaseine concentration in *Pc* relative to *Pp* due to its lower surface area: body weight ratio. A cross-section of the preserved worm showed that it was flattened such that the distance from the dorsal to the ventral surface was only 25–30% of its width. Since *Pc* is much less cylindrical than *Pp*, its cross-section being more like an ellipse, calculations based on a cylindrical shape are only rough approximations and are only meant to illustrate how body shape might be a key determinant of overall body anabaseine levels.

**Figure 6:**
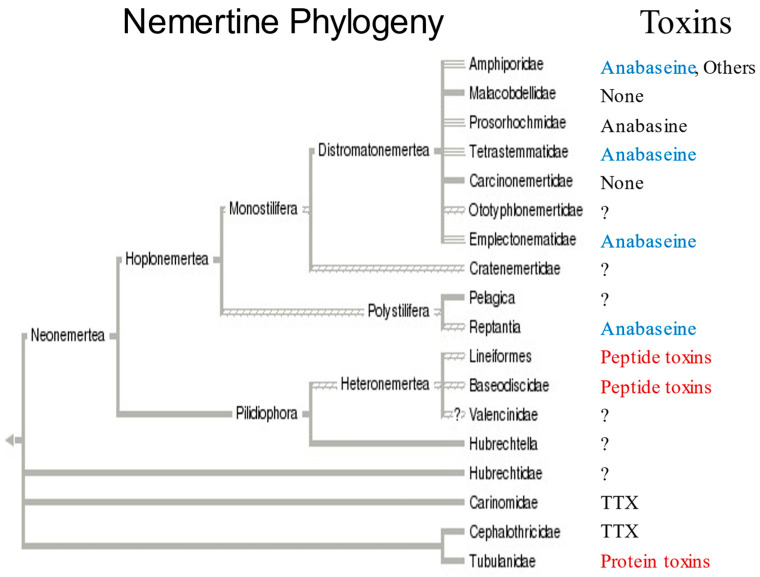
Known occurrence of toxins in nemertean taxa, based on a proposed phylogenetic tree (adapted from [16]). Major families (Amphiporidae, etc.) are listed on the right side of the tree. Currently known nemertean toxins or types of toxins are listed next to the families in which they have been found. Blue color indicates presence of anabaseine; Red color indicates peptide toxins have been found in the two other major taxa. Pilidiophora were previously called Heteronemertea and the complex comprising Hubrechtidae, Carinomidae, Cephalothricidae and Tubulanidae were called Paleonemertea. One species (*Amphiporus angulatus*) belonging to family Amphiporidae is known to have >15 pyridine alkaloids [Kem et al., in preparation]. TTX has been found in certain species [27,28,29].

A current phylogeny [16] of nemerteans is shown in Figure 6 along with the known presence of toxins in some families. The pilidiophorans and paleonemerteans shown in the bottom half of the tree frequently contain peptide neurotoxins and cytolysins ([23,24,25,26,27]; Kem et al., in preparation). Tetrodotoxin (TTX), a bacterially synthesized guanidinium neurotoxin which blocks voltage-gated sodium channels and is found in many animals including pufferfish, the blue-ringed octopus and newts, has also been found in some paleonemertines [27,28,29]. 

While the mineralized stylets of monostiliferan nemerteans have been studied in considerable detail by Stricker and colleagues [30,31,32], no electron microscopic or elemental analyses of polystiliferans have been reported. It would be of interest to determine if polystiliferan stylets, like monostiliferan stylets, are composed of the same mineral, amorphous calcium phosphate [32]. Perhaps such information can provide some basis for determining whether both forms of stylets evolved from a common primordial stylet or mineral deposit. We suggest that acquisition of pyridyl alkaloid toxins like anabaseine occurred first and mineralized deposits in the proboscis epithelium were elaborated later as a means of enhancing penetration of venom through the integuments of prey and potential predators. 

While the prey and predators of polystiliferans are still largely unknown, available data suggest that arthropods are a major prey for at least some pelagic polystiliferans [33,34]. Thus, it is possible that compounds with insecticidal activity (besides anabaseine) may be found in these worms.

## 4. Concluding Remarks

The primary conclusion of this paper, that anabaseine is found in a polystiliferan hoplonemertean, suggests that the biosynthetic mechanism for producing this alkaloid was developed before the divergent evolution of the polystiliferan and monostiliferan branches of Hoplonemertea. Analyses of other polystiliferan species can reveal how generally this alkaloid is distributed within this order. It is likely that future studies of other polystiliferans as well as monostyliferan nemerteans will lead to the discovery of novel natural products of potential human use. 

## 5. Materials and Methods

### 5.1. Animal Collection

Two living *Pc* worms were collected by a diver at 18 meters depth along the Galician (northwestern) Atlantic coast of Spain. The smallest worm (0.40 g Fr Wt) was preserved in absolute EtOH. The larger worm (2.90 g Fr. Wt.) was relaxed in 7.5% MgCl_2_, which is isotonic with seawater. The head was then sliced off, allowing removal of the proboscis, which was then sliced into anterior, median (stylet bearing) and posterior regions. Each part, including the body with head (minus a 0.65 g piece for mitochondrial DNA analysis), was separately preserved in absolute EtOH (approximately 10 mL/g Fr. Wt.) The two preserved specimens were then airmailed to the Kem laboratory for analysis.

### 5.2. Alkaloid Extraction

The ethanolic preservative was removed from each worm sample and then each worm was sliced into multiple pieces and placed in a second solution of ethanol containing 1% acetic acid to enhance extraction of the alkaloids. After shaking at 5 °C overnight, the two solvent samples of each worm were pooled, passed through a glass filter and then concentrated with a rotary evaporator at room temperature to prevent loss of volatile alkaloids like 2,3′-bipyridyl and 3-methyl-2,3′-bipyridyl. The extract for each body part was then dissolved in 2.0 mL EtOH and 0.1 mL was then used for the Ehrlich reagent spectrophotometric assay of anabaseine. The remaining body (minus proboscis) extract was used to purify anabaseine by HPLC and to obtain larger amounts of the presumed DMAB-anabaseine derivative for structural analysis.

### 5.3. Purification of Pc Alkaloids

Essentially the same Stas-Otto solvent extraction procedure previously used by King [5] to isolate “amphiporine” from *Amphiporus lactifloreus* and by Kem et al. [6] to isolate anabaseine from the wandering nemertean *Paranemertes peregrina* (*Pp*) was employed. First, methylene chloride extraction of an aqueous solution (3X with 3 Vol CH_2_Cl_2_/Vol of the crude EtOH-soluble fraction in HCl acidified water, (pH < 2) conditions was employed to remove neutral and acidic lipids, etc., followed by a similar extraction of the alkalinized (pH > 11) aqueous solution with CH_2_Cl_2,_ the weakly basic alkaloids being recovered in the alkaline CH_2_Cl_2_ phase. This procedure effectively provides the alkaloids in a much purified and concentrated form suitable for chromatographic separations.

### 5.4. Tissue Anabaseine Determinations

The use of Ehrlich’s reagent to measure anabaseine has been described in detail in earlier publications [6,7,8]. Briefly a tissue sample is homogenized in absolute EtOH containing 1% concentrated HCl, the extract is centrifuged or filtered to remove insolubles, then a portion of this ethanolic solution is added directly to the Ehrlich reagent (1% DMAB and 1% HCl in EtOH) and incubated in a closed vial for at least 3 h at 70 °C After cooling to room temperature an absorbance spectrum over the wavelength range 300–550 nm is measured to determine the wavelength of peak absorbance and the actual absorbance at 490 nm. Dilutions in 1% HCl-EtOH may be necessary to accurately determine the maximum absorbance, optimally between absorbance range 0.2–2. A 3.0 ug/mL anabaseine (free base) solution in the reaction medium gives an absorbance A = 1.00 at 490 nm The wavelength of peak absorbance for anabaseine is 487 nm under the conditions of the assay. Since Ehrlich’s reagent also reacts with indoles and other compounds containing sites susceptible to electrophilic substitution, any presumed DMAB-anabaseine derivative should be isolated chromatographically and analyzed by MS and/or NMR, as in the present paper, to ascertain its identity, assuming that sufficient material is available for these analyses. For instance, the nemertean *Amphpiporus ochraceous* contains a compound related to anabaseine that also reacts with Ehrlich’s reagent, producing a derivative with slightly shorter peak absorbance wavelengths near 480 nm in 1% HCl-ethanol ([7]; Kem et al., in preparation).

### 5.5. MS and NMR Analyses 

A reverse phase HPLC-(+)ESI-MS analysis of the presumed anabaseine peak was performed on a Thermo Fisher Scientific (Waltham, MA USA) LTQ classic quadrupole ion trap mass spectrometer operated in the positive electrospray ionization mode. ESI normal MS scans were taken and data-dependent MS/MS scans were obtained on the most abundant ion of the preceding MS scan with a 4u-isolation window, 0.3 qCID, 42.5% normalized CID and 30ms CID duration. Chromatography was performed with an Agilent (Santa Clara, CA, USA) 1100 series binary pump and a Waters (Milford, MA, USA) XTerra MS C18 (2.1 mm × 150 mm; 3.5 µm) with Phenomenex (Torrance, CA, USA) C18 Security Guard Column (2 mm × 4 mm). Mobile phase A was water plus 0.2% acetic acid and mobile phase B was methanol with 0.2% acetic acid. With a flow rate of 0.15 mL/min, the gradient was 0%B (0 min) to 30%B at 15 min then to 95%B at 60 min and held for 20 min. An Agilent (Santa Clara, CA, USA) 1100 G1314A UV/Vis detector was between the HPLC column and MS and monitored the HPLC effluent at 254 nm. 

The time-of-flight high resolution mass spectrometry (TOF-HRMS) was performed on an Agilent (Santa Clara, CA, USA) 6220A time of flight HRMS interfaced to an Agilent 1100 binary HPLC. Flow injection analysis was used to introduce the sample. The TOF-HRMS was operated in the (+)ESI mode. Elemental composition of the DMAB-natural product was calculated with the Xcalibur Qual Browser Elemental Composition tool, Qual Browser, Thermo Fisher Scientific (Waltham, MA, USA) Xcalibur 2.2 SP1.48, 12 August 2011.

The proton NMR spectrum for the 300 µg natural product DMAB-derivative free base in 0.8 mL CDCl_3_ was acquired over a period of 80 minutes on a Varian Mercury 300 MHz spectrometer (Palo Alto, CA, USA) equipped with a 5 mm conventional probe. The proton NMR spectrum for a few mg of the synthetic DMAB-anabaseine free base in 0.6 mL CDCl_3_ was acquired on a Bruker Avance III-HD 600 MHz spectrometer (Billerica, Massachusetts, MA, USA) equipped with a 5 mm cryogenic probe. Chemical shift axes in both spectra were referenced to internal tetramethylsilane at 0.0 ppm. 

## Figures and Tables

**Table 1:** Assignments of the hydrogen resonances in the proton NMR spectrum of the *Pc* natural product (NP) DMAB-derivative free base. Corresponding data for the synthetic (SYN) DMAB-anabaseine free base is included for comparison. Both spectra were recorded in CDCl_3_. The atoms in the heterocyclic rings are, starting with the N atom: 1’–6’ in the pyridyl ring, 1–6 in the tetrahydropyridyl ring. Numbering of the hydrogens in the dimethylaminobenzylidene moiety is as follows: hydrogen 7 is the connecting methine group, 9 and 12 are hydrogens on the two ortho-carbons and 10 and 14 are hydrogens attached to the meta-carbons.

H Position	Number	Chemical Shift ^1^		Multiplicity ^2^	Coupling, SYN
		NP	SYN	NP = SYN	J, Herz
2’	1	8.74	8.73	dd	2.3, 0.8
4’	1	7.81	7.81	dt	7.8, 1.9
5’	1	7.32	7.32	ddd	7.8, 4.9, 0.8
6’	1	8.62	8.62	dd	4.9, 1.7
4	2	2.87	2.87	td	6.7, 1.8
5	2	1.83	1.83	qnt	6.0
6	2	3.82	3.83	t	5.5
7	1	6.55	6.54	br t	1.7
9&12	2	7.23	7.24	m~brd	8.9
10&14	2	6.68	6.67	m~brd	8.9
11-N(CH_3_)_2_	6	2.99	2.99	s	_

^1^ Chemical shifts are in ∂-ppm from internal tetramethylsilane at 0.0-ppm. ^2^ Multiplicities: s singlet, d doublet, t triplet, qnt quintet, m multiplet, br broad.

**Table 2:** Anabaseine (Ae) distribution in the large *Paradrepanophoros crassus* specimen. Data obtained from the single dissected *Pc* specimen are compared with previously published estimates [7] for *Paranemertes peregrina (Pp)*.

Tissue	*Paradrepanophoros crassus* (Polystiliferan)			*Paranemertes peregrina* (Monostiliferan)		
Body Part	[Ae] ^1^ µg/g	Fr Wt ^2^ g	Total Ae %	[Ae] µg/g	Fr Wt g	Total Ae %
Body (-Prob.)	888	2.656	97.7	2420	0.214	69
Anterior Prob	245	0.196	2.00	10,800	0.0182	27
Median Prob	44.4	0.027	0.05	7.2	0.003	0.76
Posterior Prob	288	0.025	0.30	3.2	0.0063	0.2

^1^ [Ae] = Ae Concentration; ^2^ Fr Wt = Fresh weight.

## Data Availability

Not applicable.
